# Flash glucose monitoring system help reduce the frequency of hypoglycemia and hypoglycemic fear behavior in type 1 diabetes patients

**DOI:** 10.3389/fendo.2024.1464755

**Published:** 2024-11-12

**Authors:** Lining Dong, Junxian Li, Yanyun Hu, Ruoting Chai, Ye Zhu, Liying Zhu, Nengguang Fan, Zhijian Zhang, Jiemin Pan, Jinhua Yan, Fang Liu

**Affiliations:** ^1^ Department of Endocrinology and Metabolism, Shanghai General Hospital, Shanghai Jiao Tong University, School of Medicine, Shanghai, China; ^2^ Shanghai Key Laboratory of Diabetes, Department of Endocrinology & Metabolism, Shanghai Jiao-Tong University Affiliated Sixth People’s Hospital, Shanghai Clinical Medical Center of Diabetes, Shanghai Key Clinical Center of Metabolic Diseases, Shanghai Institute for Diabetes, Shanghai, China; ^3^ Department of Endocrinology & Metabolism, The Third Affiliated Hospital of Sun Yat-Sen University, Guangdong, China

**Keywords:** type 1 diabetes, hypoglycemia, flash glucose monitoring system, continuous glucose monitoring system, glucose coefficient of variation

## Abstract

**Objective:**

Hypoglycemia represents a serious acute complication in individuals with type 1 diabetes mellitus (T1DM). In order to more effectively identify and discriminate the occurrence of hypoglycemic events in patients with T1DM, this study aims to evaluate the impact of two distinct glucose monitoring systems—Flash Glucose Monitoring (FGM) and Continuous Glucose Monitoring (CGM)—on the management of blood glucose levels and the emotional responses associated with hypoglycemic episodes in individuals with T1DM.

**Method:**

In this study, a total of 113 patients with type 1 diabetes mellitus were enrolled and allocated to two groups for the implementation of Glucose Monitoring Systems (GMS). The groups consisted of the FreeStyle Libre group (FGM, n=56) and the ipro2 group (CGM, n=57). Participants in both groups utilized GMS at least biannually and completed a set of three questionnaires: the Diabetes Monitoring and Treatment Satisfaction Questionnaire (DMTSQ), the Diabetes Specific Quality of Life (DQOL), and the Chinese Version of the Hypoglycemia Fear Survey II (CFHSII). Clinical data, CGM metrics, and questionnaire scores were collected at the initial visit and after a one-year follow-up period.

**Results:**

The glucose coefficient of variation (GCV) and the standard deviation of blood glucose (SDBG) were independently associated with Time Below Range (TBR). Specifically, GCV could predict TBR ≥12%, with a cut-off point of 40.55. This yielded a specificity of 88.10% and a sensitivity of 68.18% in the overall patient population. For the FreeStyle Libre group and the iPro2 group, the cut-off points were 38.69 and 40.55, respectively, with specificities of 0.74 and 0.92, and sensitivities of 0.73 and 0.86, respectively. In the FreeStyle Libre group, where the frequency of use was greater than or equal to five times per year, the hypoglycemic episodes (time/month) and CHFSII-B scores were significantly reduced at follow-up compared to baseline (7.80 ± 10.25 vs 13.95 ± 14.87; 27.37 ± 11.05 vs 38.90 ± 21.61, respectively, all P <0.05).

**Conclusion:**

The utilization of multiple Flash Glucose Monitoring (FGM) implementations proved to be valuable in discriminating the occurrence of hypoglycemia and mitigating the fear of hypoglycemic episodes in patients with type 1 diabetes. Within the parameters of Glucose Monitoring Systems (GMS), the glucose glycemic variability (GCV) was identified as a predictive factor for the risk of severe hypoglycemia (TBR > 12%). The optimal cut-off point for GCV was determined to be 40.55.

## 1 Introduction

Type 1 diabetes mellitus (T1DM) is an autoimmune disorder. According to the most recent nationwide population-based registry study, the estimated incidence of T1DM per 100,000 person-years across all age groups in China is 1.01 (1). Hypoglycemia represents an acute complication of T1DM, leading to both short-term and long-term physical adverse outcomes. The blood glucose management of patients with T1DM has an impact on the risk of complications (2). For example, poor glycemic control is associated with cardiac autoimmunity and may increase the risk of cardiovascular disease (CVD) (3) and fractures (4) in T1DM.Furthermore, it exerts a significant impact on psychosocial well-being, particularly by augmenting the fear of hypoglycemic episodes (5, 6). The adoption of higher hemoglobin A1c (HbA1c) targets is thought to diminish the risk of hypoglycemia (7). However, HbA1c reflects an average blood glucose level over the preceding 2-3 months and has a limited association with glycemic variability (8) and hypoglycemia (9). Conventional self-monitoring of blood glucose (SMBG) has been deemed inconvenient and insensitive for T1DM patients in the context of hypoglycemia prevention (10).

Continuous Glucose Monitoring (CGM) systems offer a convenient means of automatically recording interstitial fluid glucose concentrations at intervals of 5 to 15 minutes over several days. Previous evidence (11, 12) has demonstrated the benefits of CGM systems in glycemic control and the reduction of hypoglycemic episodes. Moreover, metrics of glycemic variability (GV) are emerging as valuable tools for the prediction of diabetic complications. For instance, Lu (13) reported that patients with more advanced diabetic retinopathy (DR) exhibited significantly reduced time spent within the glucose target range (TIR). Bragd et al (14) found that standard deviation of blood glucose (SDBG) not only showed significance in predicting the incidence of peripheral neuropathy, but also was a highly significant predictor of hypoglycemic unawareness in type 1 diabetes. In addition, Toschi et al (15) and Zhu et al (16)found that glucose coefficient of variation (GCV) from CGMs can identify individuals at higher risk for hypoglycemia compared with HbA1c in T1DM.

Time Below Range (TBR) represents the percentage of time per day that blood glucose levels are below 3.9 mmol/L, providing crucial insights into the duration of hypoglycemic episodes. Type 1 diabetes mellitus (T1DM) is characterized by significant glycemic fluctuations, making individuals with this condition more susceptible to hypoglycemia compared to those with type 2 diabetes. Flash Glucose Monitoring (FGM) systems, such as the FreeStyle Libre, are novel glucose monitoring technologies that provide continuous glucose data for up to 14 days per sensor wear, thereby enhancing the quality of life and satisfaction with diabetes monitoring and treatment among patients (17).

In light of these advancements, the present study aimed to investigate the association between TBR and glycemic variability (GV) in Chinese patients with T1DM by utilizing various Glucose Monitoring Systems (GMS). Additionally, the study sought to explore the impact of FGM on glycemic control and the fear of hypoglycemia after approximately one year of follow-up.

## 2 Methods

### 2.1 Study population

**Figure 1:**
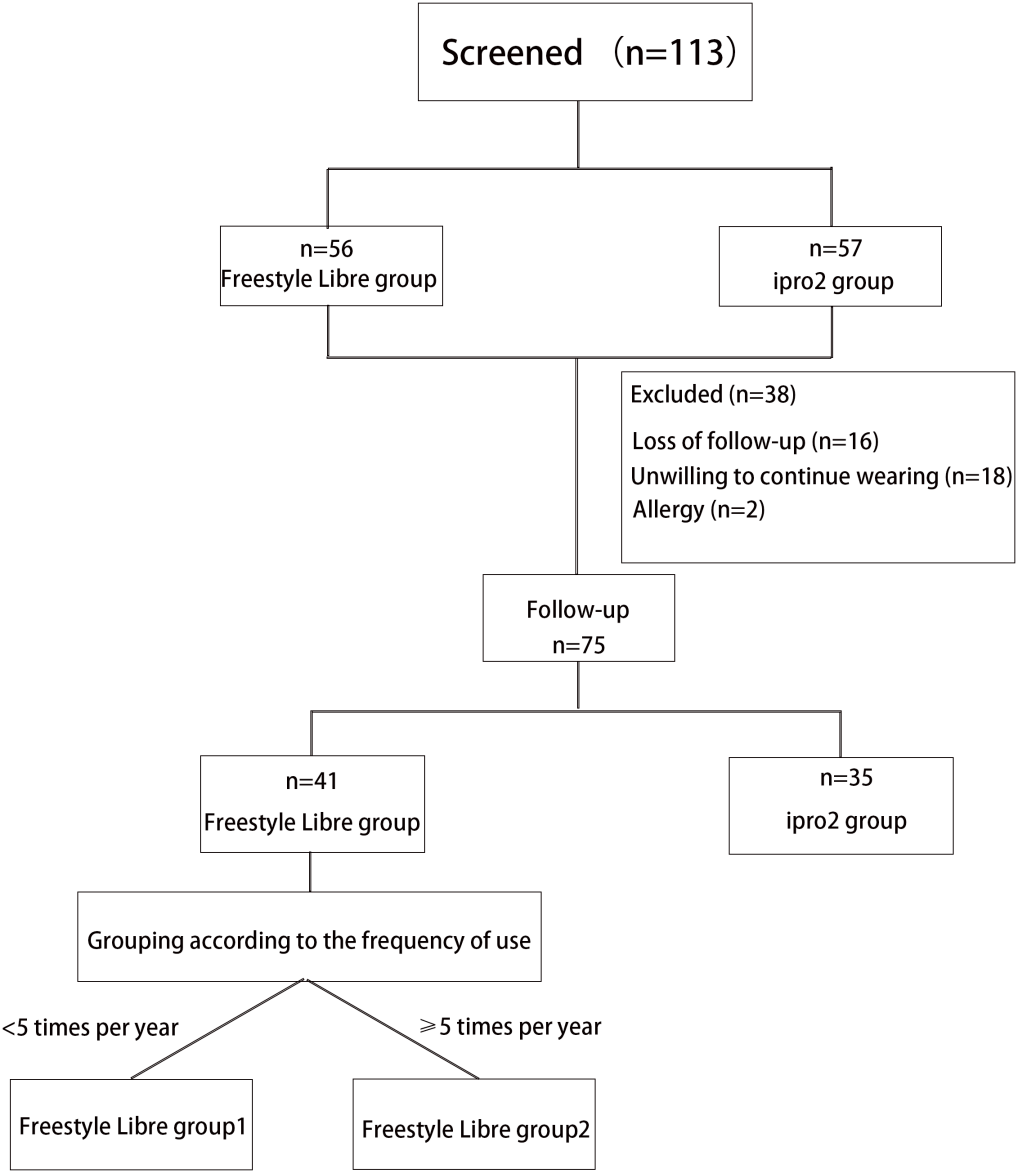
Flowchart of T1D patients recruitment and follow-up. A total of 113 patients with type 1 diabetes (T1D) were enrolled in this study following a screening process. Of these, 56 patients wore the FreeStyle Libre system, and 57 patients wore the iPro2 system. However, 38 patients were excluded from the study due to skin allergies (n=4), reluctance to continue wearing the device (n=18), and loss to follow-up (n=16). Consequently, 75 patients were followed up for approximately 1 year. Among these, 41 participants wore the FreeStyle Libre system, and 34 participants wore the iPro2 system. The FreeStyle Libre group was further divided into two subgroups based on wearing frequency: Group 1 (Worn less than five times per year) and Group 2 (Worn at least five times per year).

This study was conducted as a non-masked controlled trial, with participants, investigators, and study staff not being blinded to group allocation. A total of 120 patients with type 1 diabetes mellitus (T1DM) were recruited, and 113 of these were ultimately included in the study (**Figure 1**). The participants were recruited from the Departments of Endocrinology and Metabolism at Shanghai General Hospital, affiliated with Shanghai Jiao-Tong University School of Medicine; the Shanghai Jiao Tong University School of Medicine Affiliated Sixth People’s Hospital; and the Third Affiliated Hospital of Sun Yat-sen University. Recruitment and follow-up of patients occurred from March 2018 to May 2021.Inclusion criteria for participation were as follows: willingness to participate in the study; a confirmed diagnosis of T1DM with a history of insulin use for at least 3 months; an age of 6 years or older; the technical proficiency to utilize a glucose monitoring system; and agreement to perform self-monitoring of blood glucose (SMBG) at least three times daily. Exclusion criteria included the following: a current diagnosis of hypoglycemia unawareness; a history of diabetic ketoacidosis or myocardial infarction within the preceding 6 months; known allergy to medical-grade adhesives; use of continuous glucose monitoring within the previous 4 months; pregnancy or intention to become pregnant; and receipt of oral steroid therapy.

The iPro2 group (n=57) utilized a retrospective Continuous Glucose Monitoring (CGM) system (Medtronic Inc., Northridge, CA) for a period of three consecutive days. The FreeStyle Libre group (n=56) employed the FreeStyle Libre system (Abbott Diabetes Care, Witney, UK) for a duration of fourteen consecutive days. Both groups were required to perform Glucose Monitoring Systems (GMS) assessments at least twice annually. Following a one-year follow-up period, a total of 74 patients completed the study.

The study protocol was ethically approved by the Institutional Review Board (IRB) of Shanghai Jiao Tong University Affiliated Sixth People’s Hospital, in compliance with the ethical principles outlined in the Declaration of Helsinki. Informed written consent was obtained from all participants prior to their inclusion in the study. This trial was registered with the Chinese Clinical Trial Registry (ClinicalTrials.gov) under the registration number ChiCTR1900025495, ensuring transparency and accountability in clinical research.

### 2.2 Clinical parameters collection

Prior to the commencement of Glucose Monitoring System (GMS) monitoring, comprehensive baseline data was collected from all subjects, encompassing demographic information such as age, sex, duration of diabetes, presence of diabetes-related complications, and details of insulin therapy. Additionally, anthropometric measurements were recorded, including height, weight, systolic and diastolic blood pressure. Body Mass Index (BMI) was calculated using the formula BMI = body weight (in kg)/height2 (in m2). A range of laboratory assessments was conducted, which included measurements of fasting plasma glucose, fasting C-peptide levels, HbA1c, a comprehensive lipid profile, and urine analysis.

### 2.3 CGMS parameters collection

Following the monitoring period, a suite of glycemic metrics was calculated, including Mean Blood Glucose (MBG), Time in Range (TIR), Time below Range (TBR), Time above Range (TAR), and measures of glycemic variability (GV). These metrics were calculated as follows:

- MBG: Defined as the average blood glucose level across all measured values.
- TIR: Represented the percentage of time during a 24-hour period that blood glucose levels remained within the target range of 3.9–10.0 mmol/L.
- TBR: Indicated the percentage of time during a 24-hour period that blood glucose levels were below 3.9 mmol/L.
- TAR: Measured the percentage of time during a 24-hour period that blood glucose levels exceeded 10.0 mmol/L.
- GV: This was quantified by metrics such as the standard deviation of blood glucose (SDBG), the glucose coefficient of variation (GCV), and the largest amplitude of glycemic excursion (LAGE). GCV was calculated by dividing SDBG by MBG. LAGE was defined as the difference between the maximum and minimum blood glucose levels observed during the monitoring period.

### 2.4 Questionnaire collection

Upon completion of the baseline and follow-up visits, during which the Glucose Monitoring System (GMS) was employed, patients were required to complete three questionnaires: the Diabetes Monitoring and Treatment Satisfaction Questionnaire (DMTSQ), the Diabetes Specific Quality of Life (DQOL), and the Chinese Version of the Hypoglycemia Fear Survey II (CHFSII). The CHFSII encompasses two subscales: the Behavior (CHFSII-B) and the Worry (CHFSII-W) subscales (18).

### 2.5 Statistical analysis

Data were presented as mean ± standard deviation (SD) for continuous variables and as percentages (%) for categorical variables. Comparisons between groups were conducted using the Chi-square test and the Mann-Whitney U test for categorical variables, and the Student’s t test for continuous variables. The relationship between glycemic variability (GV) metrics and baseline characteristics was assessed using multiple stepwise linear regression analysis. The Receiver Operating Characteristic (ROC) curve was utilized to determine the cut-off value of the glucose coefficient of variation (GCV) for identifying the occurrence of abnormal Time Below Range (TBR) values (≥12%). Differences between baseline and follow-up data were evaluated using the Paired sample t test for continuous variables and the McNemar’s test for categorical variables. All statistical analyses were performed using SPSS 26.0 (SPSS Inc., Chicago, IL) and GraphPad Prism 9.0. A two-sided P value < 0.05 was considered statistically significant.

## 3 Results

### 3.1 Participants characteristics

A total of 113 patients with type 1 diabetes mellitus (T1DM) were included in the study, with a gender distribution of 54 males and 59 females. The participants had a mean age of 35.7 ± 16.3 years and had been diagnosed with diabetes for a duration of 3.136 ± 3.032 years. The mean daily insulin dose was 31.59 ± 14.24 units, and the mean monthly frequency of hypoglycemic episodes was 7.531 ± 12.055. Fasting plasma glucose (FPG) and hemoglobin A1c (HbA1c) levels were 8.52 ± 3.60 mmol/L and 7.58 ± 1.66%, respectively. Time in Range (TIR), Time above Range (TAR), Time below Range (TBR), Standard Deviation of Blood Glucose (SDBG), and Glucose Coefficient of Variation (GCV) were measured at 63.43 ± 22.50%, 29.86 ± 23.29%, 6.72 ± 9.79%, 2.95 ± 1.23, and 34.05 ± 11.14, respectively. Scores on the Chinese Version Hypoglycemia Fear Survey II (CHFSII) subscales Behavior (CHFSII-B) and Worry (CHFSII-W), as well as the Diabetes Specific Quality of Life (DQOL) and Diabetes Monitoring and Treatment Satisfaction Questionnaire (DMTSQ), were 25.94 ± 15.59, 14.20 ± 9.16, 99.39 ± 25.06, and 60.72 ± 18.40, respectively (**Table 1**).

**Table 1:** Clinical characteristics and questionnaire scores of type 1 diabetes participants.

	All participants (*n* = 113)	Freestyle Libre group (n=56)	ipro2 group (n=57)	*p*
Male sex(%)	54,47.8%	25,44.6%	29,50.9%	0.509
Age (years)	35.7 ± 16.3	31.9 ± 17.4	39.4 ± 14.4	0.014
BMI (kg/m^2^)	20.920 ± 2.984	20.322 ± 3.403	21.497 ± 2.406	0.037
SBP (mmHg)	113.443 ± 11.877	110.357 ± 10.949	116.474 ± 12.064	0.006
DBP (mmHg)	70.204 ± 9.163	68.161 ± 8.043	72.211 ± 9.805	0.018
FPG (mmol/l)	8.515 ± 3.604	7.986 ± 2.989	8.998 ± 4.051	0.144
PPG (mmol/l)	10.919 ± 4.915	9.839 ± 4.724	11.976 ± 4.920	0.040
Fasting C-peptide (ng/ml)	0.483 ± 0.591	0.503 ± 0.664	0.465 ± 0.520	0.744
TG (mmol/l)	0.788 ± 0.578	0.750 ± 0.319	0.822 ± 0.740	0.537
TC (mmol/l)	4.685 ± 0.833	4.624 ± 0.714	4.740 ± 0.932	0.491
HDL-C (mmol/l)	1.607 ± 0.443	1.593 ± 0.439	1.621 ± 0.450	0.750
LDL-C (mmol/l)	2.500 ± 0.684	2.389 ± 0.608	2.600 ± 0.738	0.125
Duration of diabetes (years)	3.136 ± 3.032	2.555 ± 2.052	3.707 ± 3.686	0.043
HbA1C (%)	7.581 ± 1.664	7.255 ± 1.433	7.907 ± 1.821	0.039
Hypoglycemia times (per month)	7.531±12.055	10.214 ± 15.279	4.895 ± 6.863	0.018
Insulin use dosage (units)	31.589 ± 14.244	29.535 ± 13.972	33.608 ± 14.342	0.129
Insulin use duration (years)	2.251 ± 2.609	1.953 ± 2.016	2.544 ± 3.073	0.230
TIR (3.9-10 mmol/L)	63.434 ± 22.501	63.714 ± 20.006	63.158 ± 24.887	0.896
TAR (>10 mmol/L)	29.858 ± 23.293	27.446 ± 22.096	32.228 ± 24.374	0.277
TBR (<3.9 mmol/L)	6.717 ± 9.787	8.839 ± 10.173	4.632 ± 8.900	0.022
MBG(mmol/L)	8.664 ± 2.357	8.320 ± 2.447	9.001 ± 2.237	0.125
SDBG(mmol/L)	2.949 ± 1.227	3.072 ± 1.358	2.843 ± 1.103	0.342
GCV(%)	34.054 ± 11.142	36.376 ± 9.165	32.059 ± 12.330	0.046
LAGE	10.435 ± 3.973	10.574 ± 3.826	10.292 ± 4.157	0.730
CHFSII-B score	25.941 ± 15.588	31.951 ± 19.034	20.341 ± 8.433	<0.001
CHFSII-W score	14.200 ± 9.157	15.439 ± 9.897	13.046 ± 8.358	0.231
DQOL score	99.386 ± 25.058	102.902 ± 23.422	95.952 ± 26.385	0.208
DMTSQ score	60.718 ± 18.402	60.135 ± 14.956	61.244 ± 21.213	0.792

CGM, Continuous glucose monitoring; BMI, body mass index; SBP, systolic blood pressure; DBP, diastolic blood pressure; FPG, fasting plasma glucose; PPG, postprandial plasma glucose; TC, total cholesterol; TG, total triglycerides; HDL-C, high-density lipoprotein cholesterol; LDL-C, low-density lipoprotein cholesterol; HbA1c, glycosylated hemoglobin A1c; TIR, Time in Range; TAR, Time above Range; TBR, Time below Range, MBG, Mean Blood Glucose; SDBG,standard deviation of blood glucose; GCV, glucose coefficient of variation; LAGE, largest amplitude of glycemic excursions; CHFSII-B/W,Chinese Version Hypoglycemia Fear Survey II- Behavior /Worry; DMTSQ, Diabetes Monitoring and Treatment Satisfaction Questionnaire; DQOL,Diabetes Specific Quality of Life.

a Data were expressed as mean ± standard deviation (SD) for continuous variables, and percentages (%) for categorical variables.

The study involved 57 patients who were implanted with the iPro2 system (retrospective Continuous Glucose Monitoring, CGM) and 56 patients who were implanted with the FreeStyle Libre system (Flash Glucose Monitoring, FGM). Significant differences were observed in Time Below Range (TBR), Glucose Coefficient of Variation (GCV), and scores on the Chinese Version Hypoglycemia Fear Survey II Behavioral subscale (CHFSII-B) between the two groups. Specifically, the TBR, GCV, and CHFSII-B scores were found to be higher in the FGM group compared to the iPro2 group (**Table 1**).

### 3.2 The associated factors of TBR

**Figure 2:**
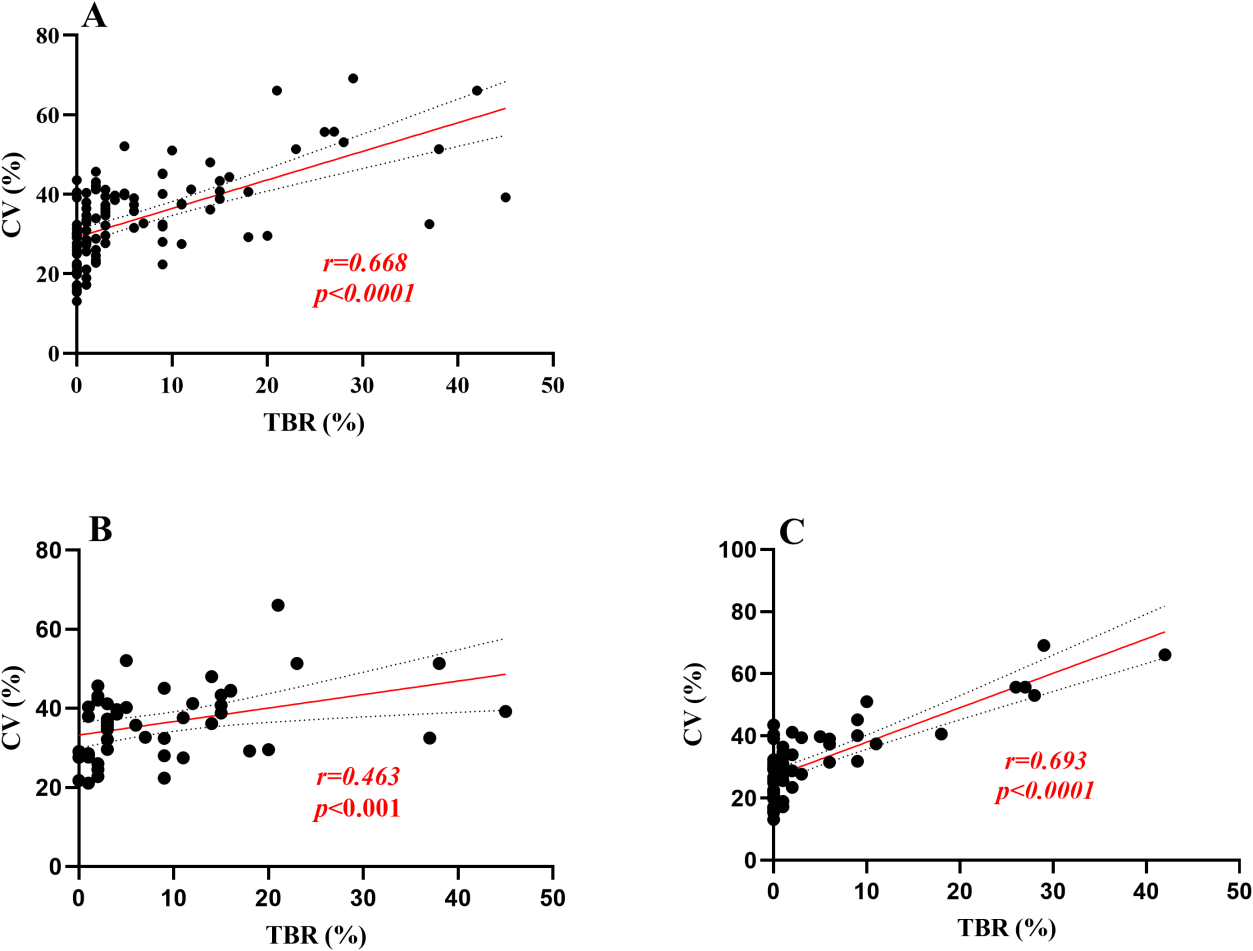
Linear regression analysis was performed for TBR and GCV. **(A)** means the correlation coefficient between TBR and GCV was 0.668 in all participants, p<0.0001;**(B, C)** means in Freestyle Libre group and Ipro2 group the correlation coefficient between TBR and GCV was 0.463,0.693, respectively, all P < 0.001].

In this study, Time Below Range (TBR) was found to be significantly correlated with Time Above Range (TAR), Mean Blood Glucose (MBG), Standard Deviation of Blood Glucose (SDBG), Glucose Coefficient of Variation (GCV), HbA1c, Fasting C-peptide levels, and scores on the Chinese Version Hypoglycemia Fear Survey II Behavioral subscale (CHFSII-B) in all patients with type 1 diabetes (**Table 2**). To further analyze the independent association factors of TBR, a multiple stepwise linear regression analysis was performed. The results indicated that GCV and SDBG were independent impact factors of TBR, after adjusting for other clinical confounding factors such as age, sex, body mass index (BMI), insulin dosage, duration of insulin use, fasting-C peptide levels, HbA1c, and other glycemic variability (GV) metrics (**Table 3**). Spearman correlation analysis revealed that GCV significantly correlated with TBR in both patient groups that wore the iPro2 and the FreeStyle Libre systems [correlation coefficients (r) = 0.693 and r = 0.463, respectively, all P < 0.001] (**Figure 2**).

**Table 2:** Correlation analysis of variables with TBR in all T1DM.

Variables	*r*	*P*
TIR (3.9-10 mmol/L)	-0.124	0.191
TAR (>10 mmol/L)	-0.234	0.012
MBG(mmol/L)	-0.450	0.000
SDBG(mmol/L)	0.234	0.016
CV(%)	0.668	0.000
LAGE	0.098	0.342
HbA1c	-0.320	0.001
FPG	-0.142	0.140
PPG	-0.157	0.140
Fasting C-peptide	-0.255	0.007
Diabetic duration	0.033	0.731
age	-0.141	0.136
BMI	-0.078	0.415
Insulin daily dose	0.052	0.585
SBP	0.058	0.540
DBP	0.035	0.712
TC	-0.102	0.316
TG	-0.130	0.199
HDL	-0.058	0.565
LDL	-0.081	0.425
Sex	0.178	0.060
CHFSII-B	0.247	0.023
CHFSII-W	0.149	0.172
DQOL	0.052	0.643
DMTSQ	0.141	0.217

BMI, body mass index; SBP, systolic blood pressure; DBP, diastolic blood pressure; FPG, fasting plasma glucose; PPG, postprandial plasma glucose; TC, total cholesterol; TG, total triglycerides; HDL-C, high-density lipoprotein cholesterol; LDL-C, low-density lipoprotein cholesterol; HbA1c, glycosylated hemoglobin A1c; TIR, Time in Range; TAR, Time above Range ; TBR, Time below Range, MBG, Mean Blood Glucose; SDBG,standard deviation of blood glucose; CV,coefficient of variation; LAGE,largest amplitude of glycemic excursions; CHFSII-B/W,Chinese Version Hypoglycemia Fear Survey II- Behavior /Worry.;DMTSQ, Diabetes Monitoring and Treatment Satisfaction Questionnaire; DQOL, Diabetes Specific Quality of Life.

**Table 3:** Linear Regression Analysis of the GCV SDBG and TBR.

Model	Parameters	B(95%CI)	Standardized β	*P*
1	GCV	1.062	1.208	0.000
	SDBG	-6.103	-0.765	0.000
2	GCV	1.472	1.675	0.000
	SDBG	-10.923	-1.369	0.000
	MBG	1.714	0.421	0.015
3	GCV	1.479	1.626	0.000
	SDBG	-8.948	-1.095	0.000
	MBG	1.762	0.430	0.016
	LAGE	-0.718	-0.282	0.015
4	GCV	1.231	1.401	0.000
	SDBG	-9.914	-1.242	0.000
	TIR	-0.209	-0.490	0.000
5	GCV	1.260	1.433	0.000
	SDBG	-8.676	-1.087	0.000
	TAR	0.106	0.257	0.012

Model 1 was adjusted for age, sex, BMI, Insulin dosage, Insulin use duration, fasting C-peptide and HbA1c.

Model 2 includes all variables in Model 1 plus MBG.

Model 3 includes all variables in Model 1 plus LAGE and MBG.

Model 4 includes all variables in Model 1 plus TIR.

Model 5 includes all variables in Model 1 plus TAR.

BMI, body mass index;; HbA1c, glycosylated hemoglobin A1c; TIR, Time in Range; TAR, Time above Range ; TBR, Time below Range, MBG, Mean Blood Glucose; SDBG,standard deviation of blood glucose; CV,coefficient of variation.

### 3.3 The predictive value of GCV for TBR≥12%

**Figure 3:**
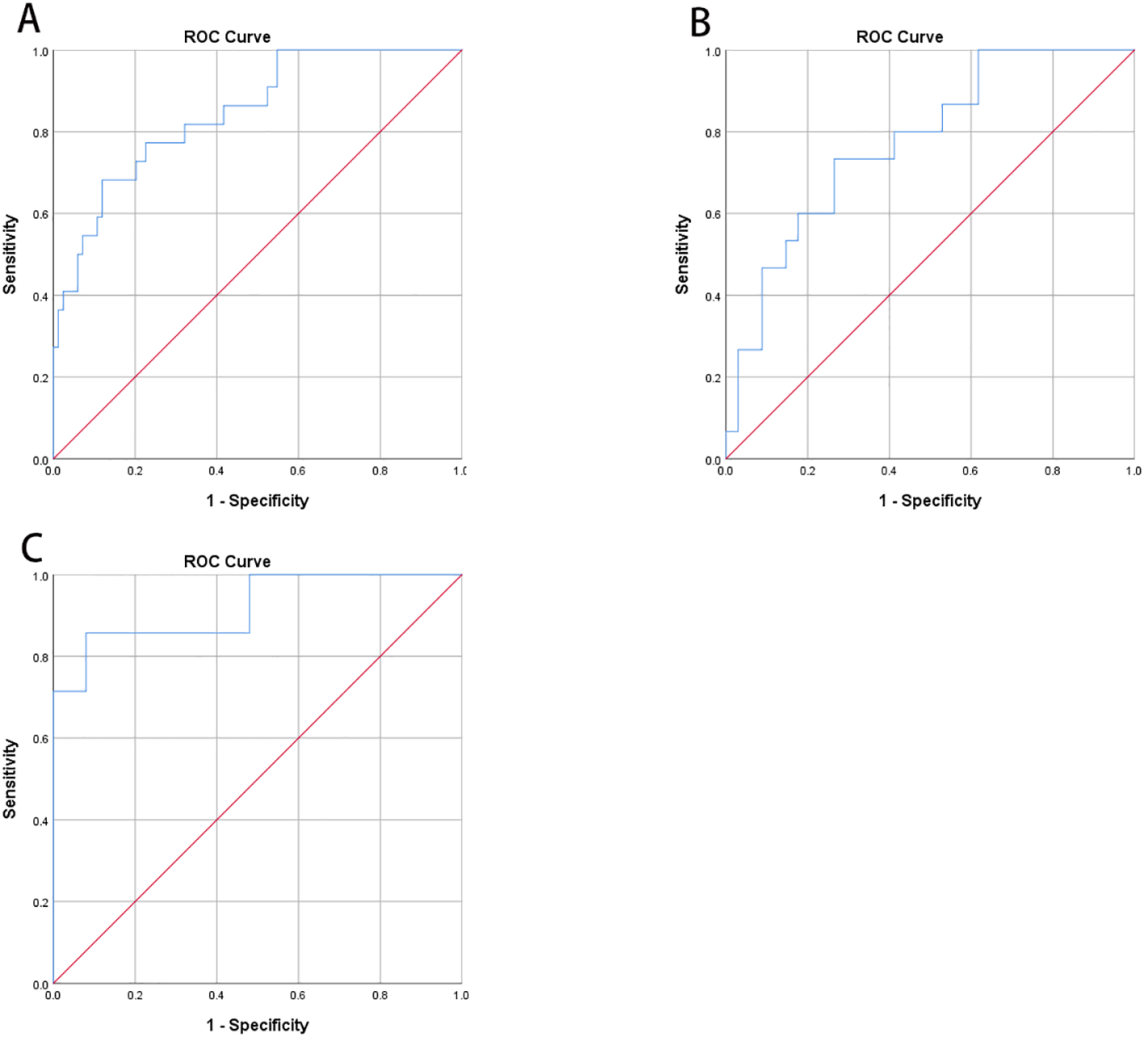
Receiver operating characteristic curve (ROC) analysis was used to know the cut-off point of GCV to predict abnormal TBR (≥12%). **(A)** In all T1DM subjects, area under the curve (AUC) of GCV was 0.847 (95%CI, 0.758-0.935; P<0.001) and the cut-off point was 40.55 with specificity 88.10% and sensitivity 68.18%, respectively. **(B)** In Freestyle Libre group, area under the curve (AUC) was 0.775 (95%CI, 0.638-0.912; P=0.002) and the cut-off point was 38.69 with specificity 73.53% and sensitivity 73.33%, respectively. **(C)** In Ipro2 group, AUC was 0.920 (95%CI, 0.793-1.000; P=0.000) the cut-off point was 40.55 with specificity 92.00% and sensitivity 85.71%, respectively (**Table 4**).

According to the Chinese clinical guidelines for continuous glucose monitoring (19), Time Below Range (TBR) ≥12% was defined as the upper threshold of unacceptable hypoglycemia. Glucose Coefficient of Variation (GCV) and Standard Deviation of Blood Glucose (SDBG) were independently correlated with the TBR level. Consequently, Receiver Operating Characteristic (ROC) curve analysis was employed to identify the cut-off value of GCV for predicting abnormal TBR (≥12%). In the overall cohort of type 1 diabetes mellitus (T1DM) subjects, the area under the curve (AUC) was 0.847 (95% Confidence Interval, 0.758-0.935; P=0.000), with a cut-off point of 40.55, yielding specificity of 88.10% and sensitivity of 68.18% (**Figure 2A**). In the FreeStyle Libre group, the AUC was 0.775 (95% CI, 0.638-0.912; P=0.002), with a cut-off point of 38.69, resulting in specificity of 73.53% and sensitivity of 73.33% (**Figure 2B**). In the iPro2 group, the AUC was 0.920 (95% CI, 0.793-1.000; P=0.000), with a cut-off point of 40.55, leading to specificity of 92.00% and sensitivity of 85.71% (**Figure 3**) (**Table 4**).

**Table 4:** The characteristics of receiver operating characteristic curve.

ROC curve (Figure 2A)			CV(%)	ROC curve (Figure 2B)			CV(%)	ROC curve (Figure 2C)			CV(%)
Sensitivity	1 - Specificity	Youden‘s index	Cut-off point	Sensitivity	1 - Specificity	Youden‘s index	Cut-off point	Sensitivity	1 - Specificity	Youden‘s index	Cut-off point
0.682	0.155	0.527	40.16	0.733	0.353	0.380	37.48	0.857	0.140	0.717	39.60
0.682	0.143	0.539	40.30	0.733	0.324	0.410	37.81	0.857	0.120	0.737	39.94
0.682	0.131	0.551	40.44	0.733	0.294	0.439	38.26	0.857	0.100	0.757	40.29
0.636	0.119	0.517	40.68	0.667	0.265	0.402	39.03	0.714	0.080	0.634	40.94
0.636	0.119	0.517	40.68	0.667	0.265	0.402	39.03	0.714	0.080	0.634	40.94
0.591	0.119	0.472	40.95	0.600	0.265	0.335	39.46	0.714	0.060	0.654	42.40
0.591	0.107	0.484	41.20	0.600	0.235	0.365	39.95	0.714	0.040	0.675	44.40

a means the cut-off point of ROC curve. CV,coefficient of variation.

### 3.4 One year follow-up of T1DM who implemented with FGM

In this study, a one-year follow-up was conducted on 41 patients who used the FreeStyle Libre system. Clinical data, along with scores from the Chinese Version Hypoglycemia Fear Survey II (CFHSII), Diabetes Specific Quality of Life (DQOL), and Diabetes Monitoring and Treatment Satisfaction Questionnaire (DMTSQ), were reassessed. Based on the frequency of FreeStyle Libre use, patients were categorized into two groups: FreeStyle Libre Group 1, with low frequency use (<5 times per year, mean ± SD of 2.43 ± 0.51), and FreeStyle Libre Group 2, with high frequency use (≥5 times per year, mean ± SD of 22.10 ± 6.11).At baseline, there were no statistically significant differences between the two groups in terms of HbA1c, age, sex, Fasting Plasma Glucose (FPG), Body Mass Index (BMI), Lipid Profile, Insulin Daily Dose, Insulin Use Duration, and scores on the CFHSII-W subscale (all P values > 0.05). However, the frequency of hypoglycemic episodes (time/month) was significantly higher in FreeStyle Libre Group 2 compared to FreeStyle Libre Group 1 at baseline (5.38 ± 6.85 vs 13.95 ± 14.87, P < 0.05). Additionally, the CFHSII-B scores were significantly lower in FreeStyle Libre Group 2 compared to FreeStyle Libre Group 1 at baseline (25.78 ± 14.77 vs 38.90 ± 21.61, P < 0.05).A comparison of the clinical characteristics and questionnaire scores between FreeStyle Libre Group 1 and FreeStyle Libre Group 2 before and after follow-up revealed that the change in DQOL scores was significantly greater in FreeStyle Libre Group 2 compared to FreeStyle Libre Group 1 (11.79 ± 26.29 vs -9.41 ± 18.21, P < 0.05) (**Table 5**).

**Table 5:** Comparison between FGM groups before and after follow-up.

	FGM group 1 (n=21)	FGM group 2 (n=20)	*P*
Baseline			
Male sex(%)	8, 38.1%	8, 40%	*0.904*
Age	34.810 ± 15.964	25.050 ± 19.484	*0.087*
BMI	20.817 ± 3.625	18.836 ± 2.770	*0.057*
HbA1c	7.424 ± 1.557	6.858 ± 1.406	*0.237*
Fasting glucose (ng/mL)	8.414 ± 3.725	7.777 ± 2.520	*0.541*
Insulin daily dose	26.638 ± 13.661	31.703 ± 12.757	*0.228*
Insulin use duration	1.788 ± 1.654	1.370 ± 1.053	*0.339*
Hypoglycemic episodes (time /month)	5.381± 6.852	13.950 ± 14.873	*0.027*
CHFSII-B scores	25.778 ± 14.767	38.895 ± 21.610	*0.039*
CHFSII-W scores	14.500± 10.314	12.842 ± 7.719	*0.582*
DQOL scores	106.611 ± 22.264	105.053 ± 24.309	*0.840*
DMTSQ scores	58.938 ± 16.909	61.737 ± 12.701	*0.580*
TG	0.890 ± 0.399	0.658 ± 0.209	*0.049*
TC	4.564 ± 0.639	4.528 ± 0.688	*0.878*
HDL-c	1.507 ± 0.424	1.597 ± 0.454	*0.557*
LDL-c	2.338 ± 0.530	2.318 ± 0.625	*0.922*
Diabetic duration	2.510 ± 1.789	2.135 ± 1.621	*0.487*
Follow-up			
Implement times per year	2.430 ± 0.507	22.100 ± 6.112	0.000
ΔFPG(ng/mL)	0.284 ± 3.180	-1.263 ± 2.840	0.144
ΔTG	0.186 ± 0.512	-0.184 ± 1.022	0.202
ΔTC	-0.125 ± 0.810	-0.203 ± 0.883	0.803
ΔHDL-c	0.126 ± 0.675	0.000 ± 0.595	0.574
ΔLDL-c	-0.251 ± 0.706	-0.178 ± 0.641	0.768
ΔHbA1c(%)	-1.016 ± 2.006	-0.141± 0.743	0.090
Δ Insulin daily dose	-5.948 ± 11.671	-6.490 ± 8.374	0.867
Δ Hypoglycemic episodes (time /month)	-0.275 ± 10.081	6.150 ± 13.072	0.090
Δ CHFSII-B scores	3.353 ± 17.150	11.526 ±18.063	0.174
Δ CHFSII-W scores	-4.675 ± 10.994	0.895 ± 6.280	0.080
Δ DQOL scores	-9.412 ± 18.211	11.790 ± 26.292	0.009
Δ DMTSQ scores	2.200 ± 7.903	0.947 ± 12.117	0.732

BMI, body mass index; FPG, fasting plasma glucose; TC, total cholesterol; TG, total triglycerides; HDL-C, high-density lipoprotein cholesterol; LDL-C, low-density lipoprotein cholesterol; HbA1c, glycosylated hemoglobin A1c; CHFSII-B/W, Chinese Version Hypoglycemia Fear Survey II- Behavior /Worry. DMTSQ, Diabetes Monitoring and Treatment Satisfaction Questionnaire; DQOL,Diabetes Specific Quality of Life. Δ means the difference between baseline and follow-up.

a. Data were expressed as mean ± standard deviation (SD) for continuous variables, and percentages (%) for categorical variables. ΔFPG = FPG baseline – FPG follow-up, and the others are the same way.

**Figure 4:**
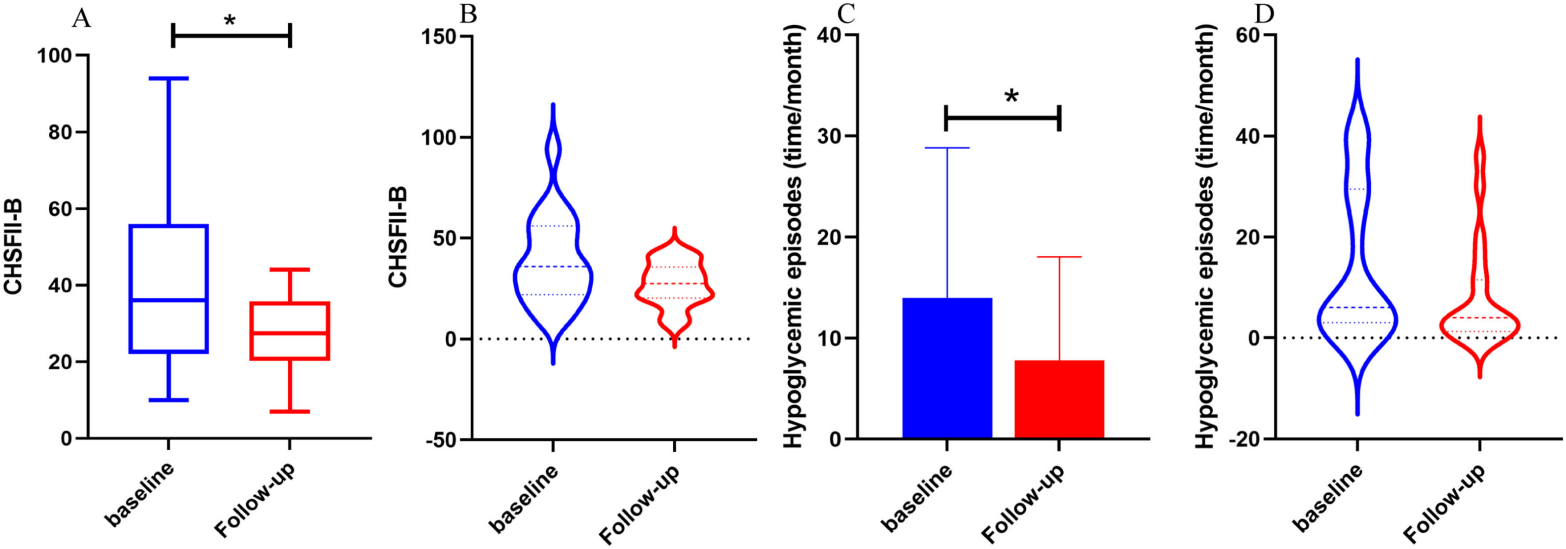
The comparison of hypoglycemic episodes, CFHSII-B between baseline and follow-up of Freestyle Libre group 2. FGM group 1 used Freestyle Libre at low frequency (<5 times per year) and FGM group 2 used Freestyle Libre at high frequency (≥ 5 times per year). In **(A, B)**, the CFHSII-B score of the follow-up group was significantly lower than baseline group, and the episodes of hypoglycemia in the follow-up group was significantly reduced compared with baseline in **(C, D)**, It can also be seen from the violin chart **(B, C)** that after follow-up, the distribution of hypoglycemia episodes (time/month) and CHFSII-B scores was more focused, and the gap between the maximum and minimum values was reduced.

We compared the follow-up clinical characteristics and questionnaire scores with the baseline data for FreeStyle Libre Group 1 and FreeStyle Libre Group 2. In FreeStyle Libre Group 2, the frequency of hypoglycemic episodes (time/month) and scores on the Chinese Version Hypoglycemia Fear Survey II Behavioral subscale (CHFSII-B) were significantly lower at follow-up compared to baseline (13.95 ± 14.87 vs 7.80 ± 10.25; 38.90 ± 21.61 vs 27.37 ± 11.05, respectively, all P<0.05). Additionally, the violin charts revealed that after follow-up, the distribution of hypoglycemic episodes (time/month) and CHFSII-B scores became more concentrated, with a narrower range between the maximum and minimum values (**Figure 4**).

In contrast, in FreeStyle Libre Group 1, there was no significant change in the frequency of hypoglycemic episodes (time/month) and CHFSII-B score. However, the Diabetes Specific Quality of Life (DQOL) score increased significantly (109.24 ± 19.87 vs 118.65 ± 23.20, P <0.05). Furthermore, the insulin daily dose increased significantly in both groups (P <0.05). Other variables, including triglycerides (TG) and fasting plasma glucose (FPG), did not show significant differences between the follow-up and baseline periods in either group (**Table 6**).

**Table 6:** Intra-group comparison before and after follow-up.

FGM group 1 (n=21)				FGM group 2 (n=20)			
category	baseline	Follow-up	*P*	category	baseline	Follow-up	*P*
FPG(ng/mL)	8.576 ± 3.842	8.292 ± 3.208	0.717	FPG(ng/mL)	7.448 ± 2.306	8.711 ± 2.202	0.085
TG	0.855 ± 0.380	0.804 ± 0.404	0.586	TG	0.661 ± 0.204	0.912 ± 0.949	0.340
TC	4.561 ± 0.670	4.686 ± 0.774	0.574	TC	4.554 ± 0.662	4.758 ± 0.619	0.372
HDL-c	1.523 ± 0.403	1.579 ± 0.452	0.648	HDL-c	1.632 ± 0.470	1.718 ± 0.436	0.494
LDL-c	2.354 ± 0.565	2.604 ± 0.717	0.207	LDL-c	2.276 ± 0.627	2.453 ± 0.587	0.285
HbA1c(%)	7.500 ± 1.557	8.516 ± 2.162	0.041	HbA1c(%)	6.635 ± 0.841	6.777 ± 0.619	0.445
Insulin daily dose	26.570 ± 14.013	32.518 ± 18.640	0.034	Insulin daily dose	31.703 ± 12.757	38.193 ± 12.873	0.003
Hypoglycemic episodes (time /month)	5.250 ± 7.003	5.525 ± 8.081	0.904	Hypoglycemic episodes (time /month)	13.950 ± 14.873	7.800 ± 10.247	0.049
CHFSII-B scores	26.353 ± 15.012	23.000 ± 7.550	0.432	CHFSII-B scores	38.895 ± 21.610	27.368 ± 11.046	0.012
CHFSII-W scores	14.412 ± 10.625	19.059 ± 12.651	0.101	CHFSII-W scores	12.842 ± 7.719	11.947 ± 6.014	0.542
DQOL	109.235 ± 19.873	118.647 ± 23.200	0.049	DQOL	105.053 ± 24.309	93.263 ± 28.276	0.066
DMTSQ	55.333 ± 9.147	53.133 ± 9.403	0.299	DMTSQ	61.737 ± 12.701	60.263 ±12.041	0.615

BMI, body mass index; FPG, fasting plasma glucose; TC, total cholesterol; TG, total triglycerides; HDL-C, high-density lipoprotein cholesterol; LDL-C, low-density lipoprotein cholesterol; HbA1c, glycosylated hemoglobin A1c; CHFSII-B/W, Chinese Version Hypoglycemia Fear Survey II- Behavior /Worry. DMTSQ, Diabetes Monitoring and Treatment Satisfaction Questionnaire; DQOL,Diabetes Specific Quality of Life.

## 4 Discussion

In this study, we identified that Glucose Coefficient of Variation (GCV) and Standard Deviation of Blood Glucose (SDBG) serve as independent risk factors for Time Below Range (TBR) in the FreeStyle Libre (FGM) and Continuous Glucose Monitoring (CGM) parameters of patients with type 1 diabetes mellitus (T1DM). A high GCV is predictive of TBR ≥12%, with the most accurate prediction achieved at a GCV of 40.55%.

Patients with T1DM who were fitted with the iPro2 and FGM systems were followed up for one year. Our findings indicate that both FGM and iPro2 contribute to the timely detection of hypoglycemic episodes in T1DM patients. Although the Receiver Operating Characteristic (ROC) curve analysis demonstrated that iPro2 was more specific and sensitive than FGM in predicting TBR using GCV, the clinical parameters and questionnaire scores of patients using FGM before and after follow-up revealed that FGM use effectively reduced the monthly frequency of hypoglycemia and hypoglycemia-related fear behaviors. Furthermore, multiple FGM wearings exhibited a more pronounced effect on hypoglycemia monitoring.

Continuous Glucose Monitoring Systems (CGMS) have been extensively employed in clinical practice, with numerous studies conducted in patients with type 1 diabetes. These studies have provided valuable insights that have informed our research. For instance, Rama et al (9) identified the Glucose Coefficient of Variation (GCV) derived from CGMS (iPro2) and Self-Monitoring of Blood Glucose (SMBG) as the most effective discriminator of hypoglycemia (<3 mmol/L), with an Area Under the Curve (AUC) of 0.88. The optimal cut-off point was 44%, yielding a sensitivity of 81.3% and a specificity of 89%, thus offering the best discrimination of subjects with hypoglycemia among those with type 1 diabetes. Bragd et al (14) found that SDBG derived from SMBG was also a highly significant predictor of hypoglycemic unawareness (P = 0.001). Saisho (20) demonstrated that the SDBG derived from CGMS data was positively correlated with the duration of hypoglycemia (<3.9 mmol/L). Torimoto (21) indicated that the GCV derived from CGMS could serve as an indicator of hypoglycemia in type 2 diabetes, with an AUC of 0.756, and the cut-off points for GCV in predicting hypoglycemia (<3.9 mmol/L) were 22%. Zhu et al (16) revealed that the GCV was strongly correlated with the percentage of time with glucose <70 mg/dL (<3.9 mmol/L) (r = 0.79; P < 0.0001) in youth with T1D.Toschi E et al (15) suggested that the GCV derived from CGMS could better identify individuals at higher risk for hypoglycemia compared to A1c alone.

Our research encompasses several unique and innovative aspects. Firstly, we compared the efficacy of two Glucose Monitoring Systems (GMS) in the recognition of hypoglycemia in patients with type 1 diabetes (T1D). The results revealed that both blood glucose monitoring systems were effective, with iPro2 demonstrating higher diagnostic sensitivity and specificity when TBR ≥ 12%. However, iPro2 is a retrospective blood glucose monitoring system, which can only reflect the blood glucose fluctuations over a span of 3 days and does not provide real-time guidance for the timely adjustment of hypoglycemic medications to reduce the duration of hypoglycemia. Secondly, our study evaluated the impact of FreeStyle Libre Monitoring (FGM) on quality of life and hypoglycemic fear behavior at baseline and follow-up. The findings indicated that the frequency of hypoglycemia was significantly reduced in the follow-up group, along with a significant decrease in the hypoglycemic fear behavior score. Rouhard et al. (22) conducted a retrospective study to assess the medium-term impact of FGM in T1DM and reported improvements in glycemic control, a slight reduction in daily insulin dose, an increase in diabetes satisfaction scores, and a decrease in hypoglycemic fear behavior scores. However, they did not observe a reduction in the frequency of hypoglycemia, particularly in well-controlled subjects. Thirdly, this study compared high-frequency FGM wear to low-frequency FGM for better glycemic control. Gomez-Peralta et al. (23) collected data on blood glucose variability, scanning frequency, and HbA1c in all Spanish individuals using Freestyle Libre to establish a Spain-specific relationship between testing frequency and glycemic parameters, and to demonstrate the associations of flash glucose monitoring with glycemic control under real-world settings. They found a positive correlation between high-frequency scanning and improved glycemic control. However, the large sample size may lead to an unfiltered sample, potentially resulting in biased outcomes. Urakami et al. (24) conducted a study on the effect of FGM on glycemic control in children and adolescents with T1D. They divided the subjects into high-frequency and low-frequency groups based on scanning frequency greater than 12 times/day, and found that scanning frequency was significantly positively correlated with TIR and negatively correlated with HbA1c. To date, more studies have focused on the influence of scanning frequency on glycemic control, while the effect of wearing frequency on glycemic control remains under-explored. Our research contributes to this field by addressing this gap.

Our study also yielded some results that diverge from previous findings. For instance, Torimoto et al. (21) reported that Mean Blood Glucose (MBG) could predict hypoglycemia in type 2 diabetes mellitus (T2DM), with ROC curve analysis indicating that the optimal cut-off point for MBG in predicting hypoglycemia was 152 mg/dL (AUC = 0.826; 95% CI: 0.753–0.900). Contrary to this, in our study, MBG was not identified as an independent risk factor for Time Below Range (TBR), thus precluding its use for predicting abnormal TBR. We hypothesize that this discrepancy may be attributed to the more stable glycemic variability in T2DM compared to T1DM, making MBG a more suitable predictor of hypoglycemia in type 2 diabetes.

In our study, while HbA1c did not decrease following the use of FreeStyle (17) Libre Monitoring (FGM), the frequency of hypoglycemia was significantly reduced. This suggests that FGM may play a pivotal role in the management of hypoglycemia but that hyperglycemia management remains inadequate. Bolinder et al. found that FGM reduced the time adults with well-controlled T1DM spent in hypoglycemia (<3.9 mmol/L [70 mg/dL]) between baseline and 6 months. Laffel et al. (25) conducted a randomized clinical trial involving adolescents and young adults and reported a slight but statistically significant decrease in mean HbA1c from 8.9% at baseline to 8.5% at 26 weeks in the CGM group, whereas there was no change in HbA1c at baseline and 26 weeks in the BGM group. Karter et al. (26) included patients with both T1DM and T2DM in their retrospective study and found that the use of real-time CGM was associated with significantly lower HbA1c levels and lower rates of emergency department visits or hospitalizations for hypoglycemia compared to non-use.

The convenience of hospital-based intravenous blood glucose monitoring is limited, and self-monitoring of blood glucose (SMBG) is less convenient than Continuous Glucose Monitoring (CGM) due to its invasive nature. Despite the discrepancy between interstitial-fluid blood glucose monitoring and intravenous blood glucose monitoring, this difference does not significantly impact blood glucose management. Kumagai et al. (27) concluded that both the FreeStyle Libre Pro (FSL-Pro) and iPro2 systems are clinically acceptable, but glucose values tended to be lower when measured using the FSL-Pro compared to the iPro2.

This study exhibits several strengths. Firstly, within the context of Continuous Glucose Monitoring (CGM) data, we identified that the Glucose Coefficient of Variation (GCV) is independently associated with Time Below Range (TBR), with a cut-off point of 40.55 for abnormal TBR (≥12%). Secondly, we discovered a positive correlation between hypoglycemia-related worry and the frequency of hypoglycemic episodes, indicating that patients with greater concern about hypoglycemia are more inclined to wear a continuous glucose monitor frequently. Thirdly, the use of the Freestyle Libre Flash Glucose Monitoring (FGM) system at high frequency has been shown to decrease the incidence of hypoglycemia and alleviate hypoglycemia-related fear behaviors.

Certainly, the present study is not without limitations. Firstly, the study cohort comprises a relatively small sample size of follow-up patients with T1DM. Therefore, further research is warranted to recruit a larger sample and extend the follow-up duration to validate the observed phenomena. Secondly, the absence of Continuous Glucose Monitoring (CGM) data from the follow-up visit precluded the analysis of changes in Time Below Range (TBR) and other glycemic variability (GV) metrics across the study groups. Thirdly, the one-year follow-up duration of this study limits its ability to assess the long-term impact of Flash Glucose Monitoring (FGM) on diabetes management.

## 5 Conclusions

In summary, the implementation of multiple Flash Glucose Monitoring (FGM) systems proved valuable in discriminating the occurrence of hypoglycemia and mitigating the fear-related behaviors in patients with type 1 diabetes. Among the Glucose Monitoring System (GMS) parameters, the Glucose Coefficient of Variation (GCV) emerged as a predictor of the risk of severe hypoglycemia (TBR > 12%), with an optimal cut-off point of 40.55. Consequently, for patients with T1DM whose blood glucose levels are prone to fluctuations, particularly adolescent patients, it is recommended to utilize real-time, non-invasive FGM systems frequently to promptly identify the risk of severe or prolonged hypoglycemia. This approach can alleviate the psychological burden associated with hypoglycemia and enhance the quality of life within the T1DM population.

## Data Availability

The original contributions presented in the study are included in the article/Supplementary Material. Further inquiries can be directed to the corresponding author.
